# Distribution of Arthritis Subtypes Among Adults With Arthritis in the United States, 2017–March 2020

**DOI:** 10.5888/pcd22.240393

**Published:** 2025-06-19

**Authors:** Anika L. Foster, Michael A. Boring, Tyler D. Lites, Janet E. Croft, Erica L. Odom, Elizabeth A. Fallon

**Affiliations:** 1 Division of Population Health, National Center for Chronic Disease Prevention and Health Promotion, Centers for Disease Control and Prevention, Atlanta, Georgia; 2 Chenega Services & Federal Solutions, LLC, Smyrna, Georgia; 3 Oak Ridge Institute for Science and Education, Oak Ridge, Tennessee; 4 Retired, Division of Population Health, National Center for Chronic Disease Prevention and Health Promotion, Centers for Disease Control and Prevention, Atlanta, Georgia

## Abstract

**Introduction:**

Arthritis is a common chronic disease, affecting an estimated 53.2 million adults (21.2%) in the US. “Arthritis” is a general term, describing over 100 conditions with different etiologies, pathogeneses, symptoms, and treatments. Few studies have examined the prevalence and distribution of arthritis subtypes in the US.

**Methods:**

We used National Health and Nutrition Examination Survey data from 2017 to March 2020 to estimate the prevalence of arthritis subtypes overall and by sociodemographic characteristics.

**Results:**

The overall prevalence of any type of diagnosed arthritis among US adults aged 20 years or older in this study was 27.9% (67.1 million). Among adults with diagnosed arthritis, osteoarthritis (49.6%, 33.2 million) was the most common arthritis subtype, followed by rheumatoid arthritis (15.8%, 10.6 million) and psoriatic arthritis (1.4%, 1.0 million). More than 1 in 10 reported some other type of unlisted arthritis (11.5%, 7.7 million), and 1 in 5 did not know their arthritis subtype (21.6%, 14.4 million). Prevalence of not knowing arthritis type was approximately 1 in 4 for adults identifying as non-Hispanic Black (26.7%) or other Hispanic (29.5%) and for adults who reported low family income (26.7%) and was approximately 1 in 3 for adults identifying as Mexican American (31.9%), having less than a high school education (31.8%), or not having health insurance (36.1%).

**Conclusion:**

Understanding arthritis type is important for improving treatment, self-management, and health outcomes associated with arthritis. Improving organizational and personal health literacy are potential strategies that may reduce the prevalence of not knowing arthritis type.

**Summary**

**What is already known about this topic?**

More than 53 million US adults have arthritis. National data on the prevalence and distribution of arthritis subtypes among adults are limited.

**What is added by this report?**

Osteoarthritis and rheumatoid arthritis remain the most common arthritis types among US adults. Among US adults with an arthritis diagnosis, approximately 1 in 5 (21.6%) did not know their arthritis type.

**What are the implications for public health practice?**

Knowing arthritis type is crucial for successfully managing the disease and preventing further damage. Using strategies to improve organizational and personal health literacy could contribute to more informed patients, thereby reducing the prevalence of not knowing arthritis type and improving health outcomes.

## Introduction

Arthritis is a common chronic disease in the US; during 2019–2021, an estimated 53.2 million (21.2%) adults reported diagnosed arthritis (1). An analysis conducted using 2010–2012 National Health Interview Survey (NHIS) data found that the number of US adults with arthritis was projected to increase to 78.4 million by 2040 (2). The term “arthritis” describes more than 100 conditions with different etiologies, pathogeneses, symptoms, and treatments. One in 4 adults with arthritis report experiencing severe joint pain (3), and nearly half (43.9%) report being limited in their everyday activities because of arthritis (4). Arthritis is also a leading driver of disability (5), costing an estimated $303.5 billion in 2013 for medical care and lost earnings (6).

Estimates during 1999–2014 showed that the most common arthritis types were osteoarthritis (OA) and rheumatoid arthritis (RA), with US population prevalences of 9.7% and 4.2%, respectively (7). Because arthritis subtypes have different etiologies, pathogeneses, symptoms, and treatments, patient awareness of their arthritis type is crucial for successfully managing the disease. Knowing one’s arthritis type is important for effective treatment planning, symptom management, and overall improvement of quality of life. In addition, up-to-date prevalence estimates for arthritis types can be used to inform policies, allocate resources, and support primary and secondary prevention programs for arthritis (7). However, studies examining the prevalence and distribution of arthritis subtypes among adults with arthritis in the US are limited. We used National Health and Nutrition Examination Survey (NHANES) data collected from 2017 to March 2020 to estimate the prevalence and population distribution of arthritis subtypes among US adults.

## Methods

NHANES is a series of cross-sectional, nationally representative surveys of the civilian, noninstitutionalized US population used to assess health-related measures through interviews and health examinations (8). Interview data from the public use NHANES 2017–March 2020 Prepandemic data files were analyzed to estimate the prevalence of arthritis subtypes. This data set was created by the National Center for Health Statistics combining incomplete data collected from the 2019–2020 survey cycle with complete data from 2017–2018 to make a nationally representative data set for 2017–March 2020 (9). For NHANES 2017–March 2020, the unweighted response rate was 51% for the interviewed sample (10). Arthritis was defined as an affirmative response to the question, “Has a doctor or other health professional ever told you that you have arthritis?” For NHANES, the arthritis question is only asked of adults aged 20 years or older. There were 9,206 respondents (99.7%) who answered this question in the interview portion of the survey. Among those who reported they had an arthritis diagnosis, the subtype was determined by the question, “Which type of arthritis was it?” and included response options of “osteoarthritis or degenerative arthritis,” “rheumatoid arthritis,” “psoriatic arthritis,” “other,” or “don’t know.” Among the 2,812 respondents who were asked this question, 2,809 respondents selected one of the response options. Age-standardized prevalence estimates and unadjusted distributions of arthritis by subtype were generated overall and by sociodemographic characteristics (sex, age group, race and ethnicity, highest attained education level, family income [percent of federal poverty level, FPL], health insurance status, and usual place for health care). Estimates were age-standardized to the 2000 projected US population by 5 age groups (20–44, 45–54, 55–64, 65–74, and ≥75 years). Age-specific estimates were calculated for the age group variable. Population counts by arthritis type were estimated by multiplying crude prevalence estimates by population totals of the US civilian noninstitutionalized population, as provided by the American Community Survey.

NHANES oversampled some racial and ethnic, age, and income groups to increase sample size and reliability of estimates for these subgroups (11). These were Hispanic; non-Hispanic Black; non-Hispanic, non-Black Asian; non-Hispanic White people and people of other races and ethnicities at or below 185% of the federal poverty level; and non-Hispanic White people and people of other races and ethnicities aged 0 to 11 years or 80 years or older. For this analysis, some groups still had an insufficient sample size to be analyzed separately and were combined into a single racial and ethnic group (ie, “other race, including multiracial”) for analytic purposes. Significant differences across demographic subgroups were assessed using *t* tests at an α level of .05. Linear trend tests were conducted for age group, family income, and education. Analyses used the interview weight (WTINTPRP) and were conducted by using SAS-callable SUDAAN (version 11; RTI International) to account for the complex survey design. This study involved secondary analysis of nationally representative survey data, which are publicly available and do not contain personal identifiers; therefore, the study was exempt from institutional review.

## Results

### Prevalence of arthritis and arthritis subtypes among all US adults

The overall crude prevalence of any type of diagnosed arthritis among US adults aged 20 years or older was 27.9% (95% CI, 25.3%–30.6%; 67.1 million) and the overall age-standardized prevalence was 24.9% (95% CI, 23.2%–26.7%). An age-standardized 12.0% (95% CI, 10.8%–13.3%) of US adults were diagnosed with OA, 4.1% (95% CI, 3.5%–4.8%) were diagnosed with RA, 0.3% (95% CI, 0.2%–0.5%) were diagnosed with psoriatic arthritis (PsA), 3.1% (95% CI, 2.4%–4.0%) were diagnosed with some other unlisted arthritis type, and 5.4% (95% CI, 4.7%–6.3%) were diagnosed with arthritis but did not know what type (Table 1).

**Table 1:** Age-Standardized Prevalence^a^ of Arthritis Subtypes^b^ Among Adults Aged 20 Years or Older, by Selected Characteristics, National Health and Nutrition Examination Survey, United States, 2017–March 2020

Characteristic	OA	RA	PsA	Other type	Don’t know type
	% (95% CI)				
**Overall**					
Crude	13.8 (12.2–15.6)	4.4 (3.7–5.2)	0.4 (0.2–0.6)	3.2 (2.4–4.2)	6.0 (5.1–7.1)
Age-standardized	12.0 (10.8–13.3)	4.1 (3.5–4.8)	0.3 (0.2–0.5)	3.1 (2.4–4.0)	5.4 (4.7–6.3)
**Sex**					
Men^c^	8.5 (7.3–10.0)	4.3 (3.4–5.4)	0.3 (0.1–0.7)	3.0 (2.2–4.1)	5.3 (4.4–6.5)
Women	15.0 (13.3–16.8)^d^	3.9 (3.3–4.6)	0.4 (0.3–0.6)	3.1 (2.3–4.3)	5.5 (4.7–6.5)
**Age, y**					
20–44^c^	3.1 (2.4–3.9)	1.9 (1.3–2.8)	0.2 (0.1–0.4)	1.8 (1.2–2.5)	2.1 (1.6–2.9)
45–54	11.1 (8.3–14.6)^d^	5.9 (4.3–8.1)^d^	0.4 (0.3–0.6)	4.4 (2.8–6.9)^d^	5.9 (4.6–7.6)^d^
55–64	22.6 (19.7–25.9)^d^	6.3 (5.0–8.0)^d^	0.9 (0.3–2.6)	3.5 (2.3–5.3)^d^	7.6 (5.7–10.0)^d^
65–74	28.4 (24.4–32.8)^d^	6.4 (4.9–8.2)^d^	0.4 (0.2–1.0)	4.9 (3.3–7.3)^d^	13.4 (11.7–15.4)^d^
≥75	34.4 (30.8–38.1)^d^	7.5 (5.2–10.8)^d^	0.1 (0.1–0.3)	5.4 (3.5–8.1)^d^	12.3 (9.9–15.0)^d^
**Race and ethnicity**					
Non-Hispanic White^c^	13.7 (12.3–15.3)	3.8 (3.0–4.9)	0.4 (0.2–0.7)	3.2 (2.3–4.4)	5.1 (4.0–6.4)
Non-Hispanic Black	8.7 (7.2–10.5)^d^	6.6 (5.4–8.1)^d^	0.4 (0.2–0.7)	3.2 (2.5–4.1)	6.7 (5.7–7.9)^d^
Non-Hispanic Asian	5.6 (4.5–7.0)^d^	2.9 (2.1–4.1)	0.2 (0.0–0.8)	1.3 (0.7–2.3)^d^	3.8 (2.5–5.7)
Mexican American	6.7 (4.8–9.3)^d^	4.8 (3.6–6.5)	0.2 (0.0–0.5)	1.6 (0.7–3.5)^d^	6.4 (4.7–8.7)
Other Hispanic	8.6 (6.8–10.9)^d^	4.4 (2.8–6.7)	0.4 (0.1–2.3)	2.9 (1.9–4.4)	8.0 (6.1–10.4)^d^
Other race, including multiracial	13.4 (10.1–17.4)	—e	0.5 (0.1–2.1)	—e	6.9 (5.1–9.2)
**Education level^f^**					
Less than high school graduate	7.4 (5.6–9.8)^d^	5.5 (4.1–7.2)^d^	0.2 (0.1–0.5)	3.4 (2.3–4.9)	7.8 (6.2–9.6)^d^
High school graduate/GED	12.8 (9.9–16.4)	4.6 (3.2–6.6)^d^	0.3 (0.1–0.7)	3.6 (2.4–5.4)^d^	5.7 (4.7–6.9)^d^
Some college	13.0 (11.6–14.5)	5.0 (3.8–6.7)^d^	0.5 (0.2–1.2)	3.6 (2.6–5.0)^d^	6.3 (5.1–7.8)^d^
College graduate or above^c^	12.3 (10.8–13.9)	2.3 (1.6–3.2)	0.2 (0.1–0.5)	2.0 (1.3–3.0)	3.6 (2.5–5.2)
**Family income, % FPL^g^**					
≤125	11.7 (10.1–13.6)	5.9 (4.9–7.0)^d^	0.4 (0.2–0.7)	3.8 (2.4–5.8)	7.3 (6.3–8.4)^d^
>125 to ≤200	13.9 (11.7–16.4)	3.8 (3.0–4.9)	0.9 (0.5–1.6)^d^	2.9 (2.0–4.2)	5.3 (3.6–7.8)
>200 to ≤400	10.9 (9.2–12.8)	3.9 (2.8–5.5)	0.2 (0.1–0.5)	2.9 (1.9–4.2)	4.6 (3.4–6.1)
>400^c^	12.1 (10.5–13.8)	3.1 (2.2–4.4)	0.2 (0.1–0.9)	2.8 (1.8–4.2)	4.3 (3.4–5.6)
**Health insurance^h^**					
Yes^c^	12.4 (11.1–13.8)	4.2 (3.6–4.9)	0.4 (0.2–0.6)	3.2 (2.4–4.2)	5.4 (4.6–6.4)
No	5.5 (3.6–8.4)^d^	3.4 (2.4–5.0)	0.1 (0.0–0.5)	1.7 (1.0–2.9)^d^	5.0 (3.1–8.1)
**Usual place for health care^i^**					
Yes^c^	12.5 (11.2–14.0)	4.5 (3.8–5.2)	0.4 (0.2–0.6)	3.3 (2.5–4.3)	5.7 (4.9–6.7)
No	7.5 (5.5–10.2)^d^	2.1 (1.5–2.9)^d^	0.1 (0.0–0.7)	1.6 (0.8–3.0)^d^	2.7 (1.8–4.2)^d^

Abbreviations: FPL, federal poverty level; GED, general educational development certificate; OA, osteoarthritis; PsA, psoriatic arthritis; RA, rheumatoid arthritis.

a Direct age-standardization to the 2000 projected US population by 5 age groups (20–44, 45–54, 55–64, 65–74, and ≥75 years) was applied. Age-specific estimates were calculated for the age-group variable. All estimates were weighted using the interview weight (WTINTPRP).

b Arthritis subtype was defined by responding to the question, “Which type of arthritis was it?” Respondents were required to select from the following options: “osteoarthritis or degenerative arthritis,” “rheumatoid arthritis,” “psoriatic arthritis,” “other,” or “don’t know.”

c Reference group for subgroup comparisons of arthritis prevalence.

d Estimates are significantly different (*P* < .05) from the reference group.

e Estimates were deemed unreliable and suppressed if the absolute CI width was greater than or equal to 0.30, or the absolute CI width was between 0.05 and 0.30 and the relative confidence interval width was more than 130%.

f Responses to the question, “What is the highest grade or level of school completed or the highest degree received?” Responses were combined into the following groups: 1) less than high school graduate; 2) high school graduate or equivalent (ie, graduated from high school or earned a general education development certificate); 3) some college (ie, some college or associate degree); and 4) college graduate or above (ie, bachelor’s, master’s, professional school, or doctoral degree).

g Family income is presented as the ratio of family income to poverty. The US Department of Health and Human Services poverty guidelines were used as the poverty measure to calculate this ratio (https://www.cdc.gov/nchs/hus/sources-definitions/poverty.htm).

h Defined by an affirmative response to the question, “Are you covered by health insurance or some other kind of health care plan? Include health insurance obtained through employment or purchased directly as well as government programs like Medicare and Medicaid that provide medical care or help pay medical bills.”

i Defined by a response of yes or “There is more than one place” to the question, “Is there a place that you usually go when you are sick or you need advice about your health?”

Women had higher age-standardized prevalence of OA (15.0%) compared with men (8.5%; *P* < .001; Table 1). Generally, OA prevalence increased with successive age groups (test for linear trend *P* < .001); adults aged 20 to 44 years (3.1%) had significantly lower prevalence of OA compared with adults aged 45 to 54 years (11.1%, *P* < .001), 55 to 64 years (22.6%, *P* < .001), 65 to 74 years (28.4%, *P* < .001), and 75 years or older (34.4%, *P* < .001). Adults who identified as non-Hispanic White had higher OA prevalence (13.7%) compared with adults who identified as non-Hispanic Black (8.7%), non-Hispanic Asian (5.6%), Mexican American (6.7%), or as other Hispanic group (8.6%; *P* < .001). Non-Hispanic White adults had similar OA prevalence to adults who identified as other race or as multiracial (13.4%). College graduates (12.3%) had higher OA prevalence compared with those with less than a high school education (7.4%; *P* = .002). Finally, adults without health insurance (5.5%) or not having a usual place for health care (7.5%) had lower OA prevalence than those with health insurance (12.4%, *P* < .001) or with a usual place for health care (12.5%, *P* < .001), respectively. No significant differences in OA prevalence were found by family income.

For RA, prevalence increased with age (test for linear trend *P* < .001) such that adults aged 20 to 44 years (1.9%) had lower RA prevalence compared with adults aged 45 to 54 years (5.9%, *P* = .001), 55 to 64 years (6.3%, *P* < .001), 65 to 74 years (6.4%, *P* < .001), and 75 years or older (7.5%, *P* < .001). Adults identifying as non-Hispanic White (3.8%) had lower RA prevalence compared only to those identifying as non-Hispanic Black (6.6%; *P* < .001), but RA prevalence did not differ significantly between non-Hispanic White and other racial and ethnic groups. College graduates (2.3%) had lower RA prevalence than those with lower levels of educational attainment (linear test for trend, *P* = .01). Adults with family income at 125% of the FPL or lower (5.9%) had higher RA prevalence than those at more than 400% of FPL (3.1%; *P* = .002; linear test for trend, *P* < .01). Finally, adults not having a usual place for health care (2.1%) had lower RA prevalence than those with a usual place for health care (4.5%, *P* < .001). No significant differences in RA prevalence were found by sex or health insurance status.

For PsA, adults living in families with incomes above 125% to 200% or less of the FPL (0.9%) had higher PsA prevalence than those above 400% of the FPL (0.2%; *P* = .04). No significant differences in PsA prevalence were found by sex, age group, racial and ethnic group, education, health insurance status, or having a usual place for health care.

Prevalence of some other unlisted type of arthritis generally increased with age (linear trend test *P* < .001): adults aged 20 to 44 years (1.8%) had lower prevalence of some other arthritis type, compared with those aged 45 to 54 years (4.4%, *P* = .007), aged 55 to 64 years (3.5%, *P* = .01), aged 65 to 74 years (4.9%, *P* = .003), and aged 75 years or older (5.4%, *P* = .004). Adults who identified as non-Hispanic White (3.2%) had a higher prevalence of some other unlisted arthritis type compared with adults who identified as non-Hispanic Asian (1.3%, *P* = .001) or Mexican American (1.6%, *P* = .02). The prevalence of some other unlisted arthritis type was not significantly different between adults who identified as non-Hispanic White (3.2%) and those who identified as non-Hispanic Black (3.2%, *P* = .98) or other Hispanic (2.9%, *P* = .75). College graduates had lower prevalence of being diagnosed with some other arthritis type (2.0%) compared with those with some college (3.6%, *P* = .01) and those with a high school education or equivalency certificate (3.6%, *P* = .03). Adults without health insurance had lower prevalence of some other arthritis type (1.7%) than those with health insurance (3.2%, *P* = .02). Finally, adults without a usual place for health care had lower prevalence of some other arthritis type (1.6%), than those with a usual place for health care (3.3%, *P* = .006).

### Prevalence of not knowing arthritis type among all US adults

Overall, 5.4% of US adults reported being diagnosed with arthritis but did not know their arthritis type. Adults aged 20 to 44 years had lower prevalence of not knowing their arthritis type (2.1%), compared with those aged 45 to 54 (5.9%, *P* < .001), aged 55 to 64 years (7.6%, *P* < .001), aged 65 to 74 years (13.4%, *P* < .001), and aged 75 years or older (12.3%, *P* < .001; test for trend, *P* < .001). Adults self-identifying as non-Hispanic Black (6.7%, *P* = .04) or other Hispanic (8.0%, *P* = .04) had higher prevalences of not knowing their arthritis type compared with adults who identified as non-Hispanic White (5.1%). No significant differences were found between adults who identified as non-Hispanic White and adults who identified as non-Hispanic Asian (3.8%, *P* = .13), Mexican American (6.4%, *P* = .22), or other race or multiracial (6.9%, *P* = .11). Prevalence of not knowing arthritis type generally increased as educational attainment decreased (linear trend test *P* < .001); college graduates or above (3.6%) had lower prevalence of not knowing their arthritis type than those with some college (6.3%, *P* < .001), a high school education or equivalent (5.7%, *P* = .01), and less than high school education (7.8%; *P* = .002). Finally, adults without a usual place for health care had lower prevalence of not knowing their arthritis type (2.7%), compared with those with a usual place for health care (5.7%; *P* < .001). No significant differences were found by health insurance or family income.

### Distribution of arthritis subtypes among US adults with an arthritis diagnosis

Among the 67.1 million US adults that have been diagnosed with arthritis, OA (crude, 49.6%; n = 33.2 million; age-standardized, 41.5%) was the most common arthritis subtype, followed by RA (crude, 15.8%; n = 10.6 million; age-standardized, 18.9%) and PsA (crude, 1.4%; n = 1.0 million; age-standardized, 1.7%). More than 1 in 10 adults with arthritis reported some other type of unlisted arthritis (crude, 11.5%, n = 7.7 million; age-standardized, 15.6%), and 1 in 5 adults with arthritis did not know their arthritis subtype (crude, 21.6%, n = 14.4 million; age-standardized, 22.3%) (Table 2).

**Table 2:** Age Standardized^a^ Distribution of Arthritis Subtypes^b^ Among Adults Aged 20 Years or Older With Doctor-Diagnosed Arthritis^c^, by Selected Characteristics, National Health and Nutrition Examination Survey, United States, 2017–March 2020

Characteristic	OA (n = 33.2 million)	RA (n = 10.6 million)	PsA (n = 1.0 million)	Other type (n = 7.7 million)	Don’t know type (n = 14.4 million)
	% (95% CI)				
**Overall**					
Crude	49.6 (45.2–54.1)	15.8 (13.8–18.1)	1.4 (0.8–2.3)	11.5 (9.2–14.3)	21.6 (18.8–24.8)
Age-standardized	41.5 (36.7–46.6)	18.9 (15.8–22.5)	1.7 (1.0–2.8)	15.6 (12.1–19.8)	22.3 (18.4–26.8)
**Sex**					
Men^d^	36.7 (29.0–45.2)	22.0 (15.6–30.2)	1.1 (0.6–2.3)	16.7 (11.4–23.8)	23.4 (17.8–30.1)
Women	44.8 (38.4–51.3)	16.6 (13.2–20.6)	2.2 (1.1–4.2)	14.7 (10.8–19.8)	21.8 (17.2–27.1)
**Age, y**					
20–44^d^	34.1 (27.4–41.5)	21.1 (15.0–28.9)	2.1 (0.9–4.6)	19.3 (14.0–26.1)	23.4 (17.5–30.5)
45–54	39.9 (31.4–49.0)	21.3 (15.7–28.3)	1.5 (1.0–2.2)	16.0 (11.2–22.2)	21.3 (16.4–27.3)
55–64	55.4 (49.0–61.6)^e^	15.5 (12.0–19.6)	—f	8.5 (5.8–12.2)^e^	18.5 (14.0–24.1)
65–74	53.0 (48.2–57.9)^e^	11.9 (9.4–14.9)^e^	0.8 (0.4–1.8)	9.2 (6.1–13.5)^e^	25.1 (21.7–28.9)
≥75	57.6 (50.4–64.5)^e^	12.6 (8.8–17.7)	0.2 (0.1–0.5)^e^	9.0 (6.1–13.2)^e^	20.5 (16.9–24.7)
**Race and ethnicity**					
Non-Hispanic White^d^	44.8 (38.8–51.4)	18.2 (13.4–24.3)	1.8 (0.9–3.6)	15.0 (11.3–19.6)	20.2 (14.8–26.8)
Non-Hispanic Black	33.0 (26.3–40.5)^e^	21.9 (17.6–27.0)	2.4 (1.0–5.8)	15.9 (11.4–21.9)	26.7 (21.2–33.0)
Non-Hispanic Asian	32.0 (23.1–42.4)^e^	23.4 (12.6–39.3)	—f	—f	21.6 (10.7–38.6)
Mexican American	30.7 (19.6–44.6)^e^	26.5 (17.6–37.8)	0.7 (0.2–2.5)	—f	31.9 (24.0–41.1)^e^
Other Hispanic	36.3 (25.8–48.3)	15.3 (8.9–25.0)	—f	—f	29.5 (20.5–40.3)
Other race, including multiracial	36.8 (27.3–47.6)	18.0 (10.6–29.0)	0.9 (0.2–3.6)	—f	21.6 (11.5–36.9)
**Education level^g^**					
Less than high school graduate	25.6 (16.1–38.1)^e^	22.0 (14.7–31.6)	0.6 (0.2–1.3)	20.1 (14.8–26.7)	31.8 (22.8–42.4)^e^
High school graduate/GED	45.1 (33.6–57.1)	14.5 (10.3–20.1)	1.5 (0.6–3.8)	14.7 (10.1–21.0)	24.1 (18.0–31.6)^e^
Some college	35.5 (31.1–40.0)^e^	22.5 (17.1–29.0)	2.2 (0.9–5.0)	16.5 (11.8–22.5)	23.4 (17.1–31.1)^e^
College graduate or above^d^	54.5 (45.4–63.2)	16.1 (10.0–24.8)	1.7 (0.6–4.4)	13.0 (7.8–20.8)	14.8 (10.2–21.0)
**Family income, % FPL^h^**					
≤125	35.1 (27.7–43.3)	22.3 (18.4–26.8)	1.7 (0.8–3.5)	14.3 (9.0–22.0)	26.7 (21.1–33.0)^e^
>125 to ≤200	47.1 (39.6–54.8)	14.6 (9.0–22.9)	—f	16.0 (11.7–21.6)	17.8 (11.9–25.8)
>200 to ≤400	41.8 (35.1–48.8)	17.7 (13.4–23.0)	—f	14.9 (7.7–27.0)	24.3 (16.1–34.9)
>400^d^	46.3 (35.7–57.1)	18.1 (10.2–30.2)	1.0 (0.3–2.8)	18.0 (10.6–28.9)	16.6 (12.4–21.9)
**Health insurance** i					
Yes^d^	42.8 (37.1–48.8)	19.1 (15.7–23.1)	1.7 (1.0–3.0)	15.6 (11.8–20.2)	20.7 (16.6–25.5)
No	27.6 (20.0–36.8)^e^	20.1 (12.5–30.7)	—f	14.8 (8.4–24.8)	36.1 (24.3–49.9)^e^
**Usual place for health care^j^**					
Yes^d^	41.5 (36.3–46.8)	19.6 (16.0–23.9)	1.9 (1.1–3.2)	15.0 (11.2–19.9)	22.0 (17.6–27.1)
No	43.1 (33.5–53.2)	14.9 (9.4–22.8)	0.6 (0.1–2.7)	17.7 (10.7–27.8)	23.7 (16.8–32.3)

Abbreviations: FPL, federal poverty level; GED, general educational development certificate; OA, osteoarthritis; PsA, psoriatic arthritis; RA, rheumatoid arthritis.

a Direct age-standardization to the 2000 projected US population by 5 age groups (20–44, 45–54, 55–64, 65–74, and ≥75 years) was applied. Age-specific estimates were calculated for the age-group variable. All estimates are weighted using the interview weight (WTINTPRP).

b Arthritis subtype was defined by responding to the question, “Which type of arthritis was it?” Respondents were required to select from the following options: “osteoarthritis or degenerative arthritis,” “rheumatoid arthritis,” “psoriatic arthritis,” “other,” or “don’t know.”

c Defined by an affirmative response to the question, “Has a doctor or other health professional ever told you that you have arthritis?”

d Reference group for subgroup comparisons of arthritis prevalence.

e Estimates are significantly different (*P* < .05) from the reference group.

f Estimates were deemed unreliable and suppressed if the absolute CI width was greater than or equal to 0.30, or the absolute CI width was between 0.05 and 0.30 and the relative confidence interval width was more than 130%.

g Responses to the question, “What is the highest grade or level of school completed or the highest degree received?” Responses were combined into the following groups: 1) less than high school graduate; 2) high school graduate or equivalent (ie, graduated from high school or earned a general education development certificate); 3) some college (ie, some college or associate degree); and 4) college graduate or above (ie, bachelor’s, master’s, professional school, or doctoral degree).

h Family income is presented as the ratio of family income to poverty. The US Department of Health and Human Services poverty guidelines were used as the poverty measure to calculate this ratio (https://www.cdc.gov/nchs/hus/sources-definitions/poverty.htm).

i Defined by an affirmative response to the question, “Are you covered by health insurance or some other kind of health care plan? Include health insurance obtained through employment or purchased directly as well as government programs like Medicare and Medicaid that provide medical care or help pay medical bills.”

j Defined by a response of “Yes” or “There is more than one place” to the question, “Is there a place that you usually go when you are sick or you need advice about your health?”

Generally, OA prevalence among adults with arthritis increased with age (test for trend, *P* < .001); adults aged 20 to 44 years (34.1%) had lower OA prevalence compared with adults aged 55 to 64 years (55.4%, *P* < .001), aged 65 to 74 years (53.0%, *P* < .001), and aged 75 years or older (57.6%, *P* < .001). OA age-standardized prevalence was higher among adults who identified as non-Hispanic White (44.8%) compared with adults who identified as non-Hispanic Black (33.0%, *P* = .008), non-Hispanic Asian (32.0%, *P* = .03), or Mexican American (30.7%, *P* = .04). No statistical differences were found in OA prevalence between adults who identified as non-Hispanic White and those who identified as other Hispanic (36.3%, *P* = .19) or other race, including multiracial (36.8%, *P* = .17). OA prevalence generally decreased with education level (test for trend, *P* = .006), with college graduates (54.5%) reporting higher OA prevalence compared with adults with some college (35.5%, *P* < .001) or adults with less than a high school education (25.6%, *P* = .001). Among US adults diagnosed with arthritis, those without health insurance (27.6%) had lower OA prevalence compared with adults with health insurance (42.8%, *P* = .02). No significant differences in OA prevalence were found by sex, family income, or having a usual place for health care.

Contrary to OA prevalence, RA prevalence among adults diagnosed with arthritis decreased with increasing age; RA prevalence was higher among adults aged 20 to 44 years (21.1%) compared with adults aged 65 to 74 years (11.9%, *P* = .007; test for trend, *P* = .003). PsA prevalence among US adults with diagnosed arthritis was higher among those aged 20 to 44 years (2.1%), compared with those aged 75 years or older (0.2%; *P* = .02; test for trend, *P* = .006). No significant differences in RA or PsA prevalences among adults with arthritis were found by sex, race and ethnicity, education level, family income, health insurance status, or having a usual place for health care.

Similar to trends for RA prevalence, prevalence of other unlisted arthritis type generally decreased with increasing age (test for trend, *P* = .002): adults aged 20 to 44 years (19.3%) had higher prevalence of some other unlisted type of arthritis compared with adults aged 55 to 64 years (8.5%, *P* < .001), 65 to 74 years (9.2%, *P* = .005), or 75 years or older (9.0%, *P* = .009). No significant differences were found in prevalence of other unlisted arthritis type among adults with arthritis by sex, race and ethnicity, education level, family income, health insurance status, or having a usual place for health care.

### Prevalence of not knowing arthritis type among US adults with an arthritis diagnosis

Among all US adults with an arthritis diagnosis, 1 in 5 did not know their arthritis type (crude prevalence, 21.6%). The age-standardized prevalence of not knowing arthritis type was about 1 in 4 adults for those self-identifying as non-Hispanic Black (26.7%), other Hispanic (29.5%), or with a family income at or below 125% of the FPL (26.7%). The prevalence of reporting not knowing arthritis type was about 1 in 3 adults for those who identified as Mexican American (31.9%), had less than a high school education (31.8%), and did not have health insurance (36.1%).

Prevalence of not knowing arthritis type was higher among adults who identified as Mexican American (31.9%) compared with those who identified as non-Hispanic White (20.2%, *P* = .03). Additionally, prevalence of not knowing arthritis type increased with decreasing educational attainment (test for trend, *P* = .006); adults with a college degree (14.8%) had lower prevalence of not knowing their arthritis type compared with adults with some college (23.4%, *P* = .02), with a high school education (24.1%, *P* = .03), or with less than a high school education (31.8%, *P* = .005). Adults with family income at or below 125% of the FPL (26.7%) had higher prevalence of not knowing their arthritis type compared with adults with family income above 400% of the FPL (16.6%, *P* = .004). Finally, adults with no health insurance had higher prevalence of not knowing their arthritis type (36.1%) compared with adults with health insurance (20.7%, *P* = .03). No differences in prevalence of not knowing arthritis type among adults with arthritis were found by sex, age, or having a usual place for health care.

## Discussion

From 2017 to March 2020, the age-standardized prevalence of having any arthritis diagnosis among US adults aged 20 years or older was 24.9%, with OA (12.0%) and RA (4.1%) being the most common types of diagnosed arthritis. Additionally, about 0.3% of US adults reported a PsA diagnosis. To our knowledge, this is the first national PsA prevalence estimate using a surveillance system representative of the noninstitutionalized US adult population (12,13).

The estimates in this study for overall arthritis and RA were similar to age-standardized estimates from NHANES 1999–2014 (24.7% and 4.1%, respectively) (7). The OA estimates from this study were slightly higher compared with age-standardized estimates from NHANES 1999–2014 (9.7%) (7). However, no statistical tests were conducted to determine if these differences were significant because the NHANES items assessing arthritis type were different across the study periods. The age-standardized prevalence estimate of any arthritis from this study using NHANES (24.9%) is similar to age-standardized estimates based on currently available Behavioral Risk Factor Surveillance System data from the Centers for Disease Control and Prevention (CDC) Chronic Disease Indicator tool (2019 = 22.8%, 2021 = 22.8%, and 2022 =23.9%) (14) and higher than previously reported age-standardized estimates from NHIS 2019–2021 (18.7%) (1). The variability of arthritis prevalence across surveys is due to methodologic differences in sampling, data collection methods, and survey items assessing arthritis (15–17). Given these differences, estimates from these studies should not be directly compared. For PsA, the prevalence estimate from this study (0.3%) is similar to previous US and global estimates (12,13,18).

This study echoes previous studies that showed a gap in knowledge of arthritis type among adults with arthritis (7). The age-standardized prevalence of US adults aged 20 years or older that had diagnosed arthritis but did not know their diagnosed arthritis type was 5.4% (14.4 million). This estimate is similar to previous estimates from NHANES during 1999–2014, which showed that prevalence of not knowing arthritis type was 5.5% in 2013–2014 (7). Although prevalence of not knowing arthritis type has remained stable from 2013–2014 to 2017–March 2020, not knowing arthritis type is inequitably distributed. Specifically, about 1 in 4 adults who identified as non-Hispanic Black (26.7%), other Hispanic (29.5%), or with a family income at or below 125% of the FPL (26.7%) reported not knowing their arthritis type. Additionally, about 1 in 3 adults who identified as Mexican American (31.9%), had less than a high school education (31.8%), and did not have health insurance (36.1%) reported not knowing their arthritis type.

Knowing arthritis type is crucial for receiving appropriate treatment, effectively managing symptoms, and understanding disease progression. Previous studies suggest that adults who did not know their arthritis type were more likely to have low personal health literacy (19,20), which is a predictor of a person’s health status and is associated with poorer health outcomes (21,22). Health literacy challenges can include problems learning about one’s medical condition because of difficulty understanding written information, lack of confidence to independently complete medical forms, and needing assistance to help read hospital or clinic materials (23). The 2003 National Assessment of Adult Literacy (NAAL) found that only 12% of Americans had proficient health literacy skills to navigate health systems, engage in medical discussions, and fully participate in managing one’s health (24). An updated version of NAAL does not exist; however, data from this report continue to play a role in informing national efforts to improve health literacy. The inclusion of the objective to “increase the health literacy of the population” in Healthy People 2030 demonstrates the ongoing commitment to improving health literacy in the United States (25).

Addressing health literacy requires a multifaceted approach involving health care providers, organizations, policymakers, and individuals. Interventions aiming to improve both organizational and personal health literacy could be used to improve health information resources, communication, informed decision-making, and access to health services. Health care providers can improve communication with patients by using the following techniques: slowing down; using plain, nonmedical language; limiting the amount of information given and repeating it; using the teach-back technique; and creating a shame-free environment (23,26,27). Furthermore, this strategy may not only improve health outcomes for patients but also help health care professionals who report low confidence in their caregiving knowledge and skills for adults with arthritis (28).

Certain nonmedical factors may indirectly affect a person’s knowledge or awareness about their arthritis type. For example, this study showed that adults with lower educational attainment had higher prevalences of not knowing their arthritis type. These findings are consistent with other studies that found that nonmedical factors, such as income and education, are associated with not knowing one’s arthritis type (29,30). Such findings are important for understanding who is at greatest risk of not knowing their arthritis type and for identifying opportunities to increase access to and timeliness of screening, accurate diagnosis, informed shared decision-making, and comprehensive treatment and self-management for a specific arthritis subtype.

The findings in this report are subject to several limitations. First, insufficient sample sizes led to suppression of prevalence estimates for “other race, including multiracial” group; therefore, inferences using this combined racial and ethnic group were not feasible. Additional research designed to estimate arthritis prevalence specifically for these groups is needed. Second, arthritis subtypes were self-reported and not validated by a health care professional. Recall bias could have led to misclassification of arthritis types, affecting the accuracy of prevalence estimates. Third, prevalence estimates may be affected by how the item assessing arthritis type is worded. For example, because the survey item assessed “diagnosed” arthritis, people with less access to health care or without a usual place for health care may not have received an arthritis diagnosis from a health care provider, despite experiencing symptoms; results from this study showing lower arthritis prevalences among those without insurance or a usual place for health care support this assertion. Taken together, it is likely that prevalence of OA is underestimated for these 2 population subgroups. Additionally, because some participants did not know their arthritis type, the prevalences of OA, RA, and PsA may be underestimated. Misreporting of arthritis type could affect the accuracy of the estimates, and to the degree that not knowing arthritis type and misreporting can vary across demographic groups, prevalence and prevalence differences could be over- or underestimated. Finally, response options were limited to OA or degenerative arthritis, RA, PsA, “other,” and “don’t know.” Therefore, national prevalence of other types of arthritis (eg, gout, spondyloarthritis) could not be estimated. Fourth, because NHANES uses a cross-sectional design, a causal relationship between sociodemographic characteristics and arthritis could not be established. Fifth, social desirability bias might play a role in self-reported characteristics. Finally, from 2017 to March 2020, the NHANES response rate was 51%, indicating potential nonresponse bias, although survey weights were applied to address this bias and improve external validity (9,31).

This study provided updated estimates of the prevalence of arthritis subtypes (OA, RA, and PsA), which can be used to monitor trends over time, inform prevention and management efforts, and prioritize national plans and resources for future health services and interventions. Results of this study highlight the importance of addressing health literacy in adults with arthritis. Implementing strategies that improve both organizational and personal health literacy has the potential to contribute to more informed patients with increased knowledge, skills, and self-confidence to manage their condition, ultimately leading to improved health outcomes.
